# Prediction of Reproductive Outcomes According to Different Serum Anti-Müllerian Hormone Levels in Females Undergoing Intracystoplasmic Sperm Injection

**DOI:** 10.1371/journal.pone.0075685

**Published:** 2013-09-17

**Authors:** Santiago Brugo Olmedo, Sabrina De Vincentiis, Evelyn De Martino, Patricia Bedecarrás, Ana María Blanco, Analía Freire, Mariano G. Buffone, Rodolfo A. Rey

**Affiliations:** 1 Centro Médico, Seremas, Buenos Aires, Argentina; 2 Centro de Investigaciones Endocrinológicas “Dr. César Bergadá” (CEDIE)CONICET – FEI – División de Endocrinología, Hospital de Niños R. Gutiérrez, Buenos Aires, Argentina; 3 Centro de Estudios Bioquímicos, Andrológicos y Ginecológicos, Buenos Aires, Argentina; 4 Instituto de Biología y Medicina Experimental (IBYME-CONICET), Buenos Aires, Argentina; Azienda Policlinico S. Orsola-Malpighi, Italy

## Abstract

**Background and aim of the study:**

Serum anti-Müllerian hormone (AMH) is a reliable marker of ovarian reserve, and it has been shown to be correlated with reproductive outcomes in grouped analyses. However, practical data is scarce for the physician and the patients to predict these outcomes in an individual couple according to serum AMH measured prior to assisted reproduction technology (ART) procedures.

**Study Design:**

To address this question, we performed an analytic observational study including 145 females undergoing intracytoplasmic sperm injection (ICSI) in a single center. Results were analyzed according to serum AMH; subgroup analyses were performed by grouping patients according to patient’s age and FSH levels.

**Results:**

The risk of cycle cancelation decreased from 64% in patients with serum AMH ≤3 pmol/L (0.42 ng/mL) to 21% with AMH ≥15 pmol/L (2.10 ng/mL). Cycle cancelation occurred in approximately two-thirds of the patients with AMH ≤ 3 pmol/L irrespective of the FSH level. However, with higher AMH values the risk of cycle cancelation decreased more significantly in patients with normal FSH. The rate of good response increased from almost null in patients with AMH ≤3 pmol/L to 61% in those with AMH ≥15 pmol/L. The positive correlation between good response and AMH was also significant, but with lower absolute rates, when patients were grouped according to their age or FSH levels. Pregnancy rate increased moderately, but significantly, from 31% with AMH ≤3 pmol/L to 35% with AMH ≥15 pmol/L.

**Conclusions:**

We provide estimates of reproductive outcomes according to individualized values of serum AMH, in general and in subgroups according to patient’s age or serum FSH, which are helpful for the clinician and the couple in their decision making about starting an assisted reproductive treatment.

## Introduction

The human ovary contains a limited population of primordial follicles, set approximately 20 weeks post-conception, when the ovary follicle reserve achieves its maximum size. Thereafter, the ovarian reserve decreases, having an impact on natural fertility which clearly declines after the age of 30 years [1,2]. The age of women giving birth is increasing worldwide due to diverse social reasons. Consequently, a growing number of couples is facing age-related infertility problems and seek medical assistance. Assisted reproductive technology (ART) outcomes have improved over the years but are limited by the ovarian response to hyperstimulation used in treatment protocols. On the other hand, ART treatments are expensive for healthcare systems or private practices and represent a stressful situation for the couple. Therefore, the necessity exists for optimization of the evaluation of the ovarian reserve in order to minimize the uncertainty of the outcome of ART procedures. Age and circulating FSH levels, which have classically been used to predict the fertility potential, lack precision under specific clinical circumstances, especially because there is a considerable variability regarding the ovarian reserve amongst women of the same age [3].

The ovarian reserve is determined by the number of primordial follicles present in the ovary and their quality. Reliable markers of oocyte quality are yet to be developed, and direct assessment of the pool of primordial follicles present in the ovaries is not feasible. However, the number of antral follicles represents a good estimator of the primordial follicle pool and, therefore, of the quantitative aspect of the ovarian reserve [4,5]. In the recent years, anti-Müllerian hormone (AMH) has been shown to represent a reliable marker of the ovarian reserve [6] and of the response to ovarian stimulation [7–10]. A member of the TGFβ superfamily initially believed to be a fetal testis hormone [11], AMH is also produced in the ovary essentially by the granulosa cells of primary and small antral follicles [12,13]. Serum AMH levels are clearly correlated with the granulosa cells mass, ranging from undetectable in normal post-menopause [14] and in Turner syndrome patients with absence of gonadal tissue [15] to very high levels in patients with polycystic ovary syndrome [16,17] or granulosa cell tumors [18]. A particular advantage of AMH as a marker of ovarian reserve is its insignificant variation during the menstrual cycle [19–21], which does not restrict AMH measurement to a particular stage of the cycle. The first in-house AMH immunoassays developed in 1990 [22–24] were replaced by commercial assays after 1998 [18,25,26]. The two different commercially available AMH assays used in the following 10 years showed clear differences in the reported levels, mainly in the low female range [27], which complicates the interpretation of the results for the clinician. Furthermore, the large number of studies assessing the performance of serum AMH as a predictor of the ovarian reserve and a prognostic factor for the outcome of ART treatments have mostly used only one cutoff value, as summarized in a recent meta-analysis [5], thus dividing the population into two groups: one below the cutoff value usually of homogeneously poor prognosis, and another one above the cutoff value which is extremely heterogeneous. Furthermore, although the effect of age and of other reproductive variables on serum AMH has been acknowledged for years, most of the studies have analyzed serum AMH in the studied samples as a whole, or after introducing complex correction factors in the statistical analysis which hamper a simple interpretation of the AMH value observed in an individual patient presenting to the clinician.

The adequate identification of the responsiveness potential specific for the serum AMH level in each patient before entering an ART treatment may be most helpful for the couple in order to decide whether to start treatment and for the clinician in the proper management regarding the stimulation protocol. The objective of this work was to provide the clinician with a reliable tool to predict the most commonly used reproductive outcomes in women undergoing intracytoplasmatic sperm injection (ICSI) before starting the procedure. To fulfill this objective we assessed the clinical value of different serum levels AMH in predicting the rates of: cycle cancelation, good response to ovarian stimulation, syngamy, cleavage, implantation and clinical pregnancy. The assessment was performed separately according to patients’ age (25-37 and 38-43 yr) and to serum FSH (normal or elevated).

## Methods

### Study subjects and design

#### Subjects

We performed an observational study, retrospectively collecting data from 145 consecutive women, aged 25 to 43 years-old, undergoing ICSI at Seremas Institute (Buenos Aires, Argentina) between August 2009 and October 2010. The study was approved by the Seremas Institutional Review Board. The need for written informed consent was waived by the Institutional Review Board because, owing to the retrospective observational design used, study results could not modify any clinical decision made at the moment of ICSI procedures.

#### Ovarian stimulation

All patients were submitted to a standard GnRH agonist protocol, and underwent controlled ovarian hyperstimulation with recombinant FSH (Gonal-F; Serono Laboratories, Switzerland). Ovulation was induced with highly purified hCG (Ovidrel, Serono). Daily FSH doses and timing of hCG administration were adjusted according to the usual criteria of follicle maturation [28]. Follicle count was performed by ultrasonography using a transvaginal probe.

#### Serum hormone levels

Serum was obtained at first visit for AMH measurement, and during ovarian stimulation on day 3 (d3) for FSH and E2 measurement and on the day of hCG administration (d-hCG) for E2 measurement. AMH was measured at the Centro de Investigaciones Endocrinológicas, Hospital Ricardo Gutiérrez (Buenos Aires, Argentina), using an ultrasensitive enzyme-linked immunoassay specific for human AMH (EIA AMH/MIS®, Immunotech, Beckman-Coulter Co., Marseilles, France, ref. A11893), recently validated by our group [29]. FSH and estradiol (E2) were measured by chemiluminescence using Access® technology (Beckman Coulter Inc.).

#### Intracytoplasmic sperm injection (ICSI)

Conventional ICSI, as previously described [30], was conducted ~5 hours post oocyte aspiration. Motile sperm were isolated using the swim up technique. Around 2 µl of sperm were placed in 7% polyvinylpyrrolidone and a sperm was injected into each oocyte using standardized techniques. The embryos were cultured in a single droplet containing 20 µl of medium and incubated at 37°C under controlled conditions (5% CO_2_, 5% O_2_ and 90% N_2_). All embryo transfers were performed 72 hours after oocyte aspiration. Since reproductive outcomes may be associated with cycle rank (i.e. the number of previous ART cycles a patient had already been submitted to) patients were included only if they were in their first three attempts.

#### Outcome measures

The main outcome measure was the absolute risk (risk rate) of cycle cancelation (number of patients with cancelled cycles divided by the total number of patients in whom a cycle was initiated), good response to ovarian stimulation (defined as ≥ 5 oocytes retrieved at the time of aspiration), syngamy, cleavage at 48 h, multinucleated embryos, implantation (defined as the number of gestational sacs observed on ultrasound) and clinical pregnancy (defined as the presence of fetal heart activity detected by ultrasound at 6 weeks) for each of the following serum AMH levels: 3, 6, 9, 12 and 15 pmol/L. Secondary outcome measures were the predictive values of: patient’s age, serum FSH and estradiol on d3, and serum estradiol and follicle count by ultrasound assessment on d-hCG.

### Statistical analyses

Data distribution was assessed for normality using the Shapiro-Wilk test. Data of the risk rates of cycle cancelation, good response to ovarian stimulation, syngamy, cleavage at 48 h, multinucleated embryos, implantation and clinical pregnancy are presented with their 95% confidence intervals. Patient’s age, basal serum AMH, FSH and estradiol, and serum estradiol and follicle count by ultrasound assessment on the day of ovulation induction by hCG administration are presented as the median and interquartile range. Comparisons between 2 groups were made using an unpaired t test, except when a non-Gaussian distribution was found, where a Mann-Whitney test was used. Areas under the ROC curves were calculated for age and hormone levels to estimate the predictive values for the rates of: cycle cancelation, good response to ovarian stimulation, syngamy, cleavage at 48 h, multinucleated embryos, implantation and clinical pregnancy. Analyses wer performed for the whole group, and independently according to age, where the study cohort was divided into 2 groups (25-37 yr and 38-43 yr), or according to FSH levels, where the study cohort was divided into 2 groups (FSH within the normal reference range and FSH above the reference range). The absolute risk values were compared using a one-sided Fisher’s exact test. The level of significance was set at *P* <0.05. All statistical analyses were performed using GraphPad Prism version 5.01 for Windows (GraphPad Software, San Diego, CA, USA).

## Results

### Cycle cancelation

**Figure 1:**
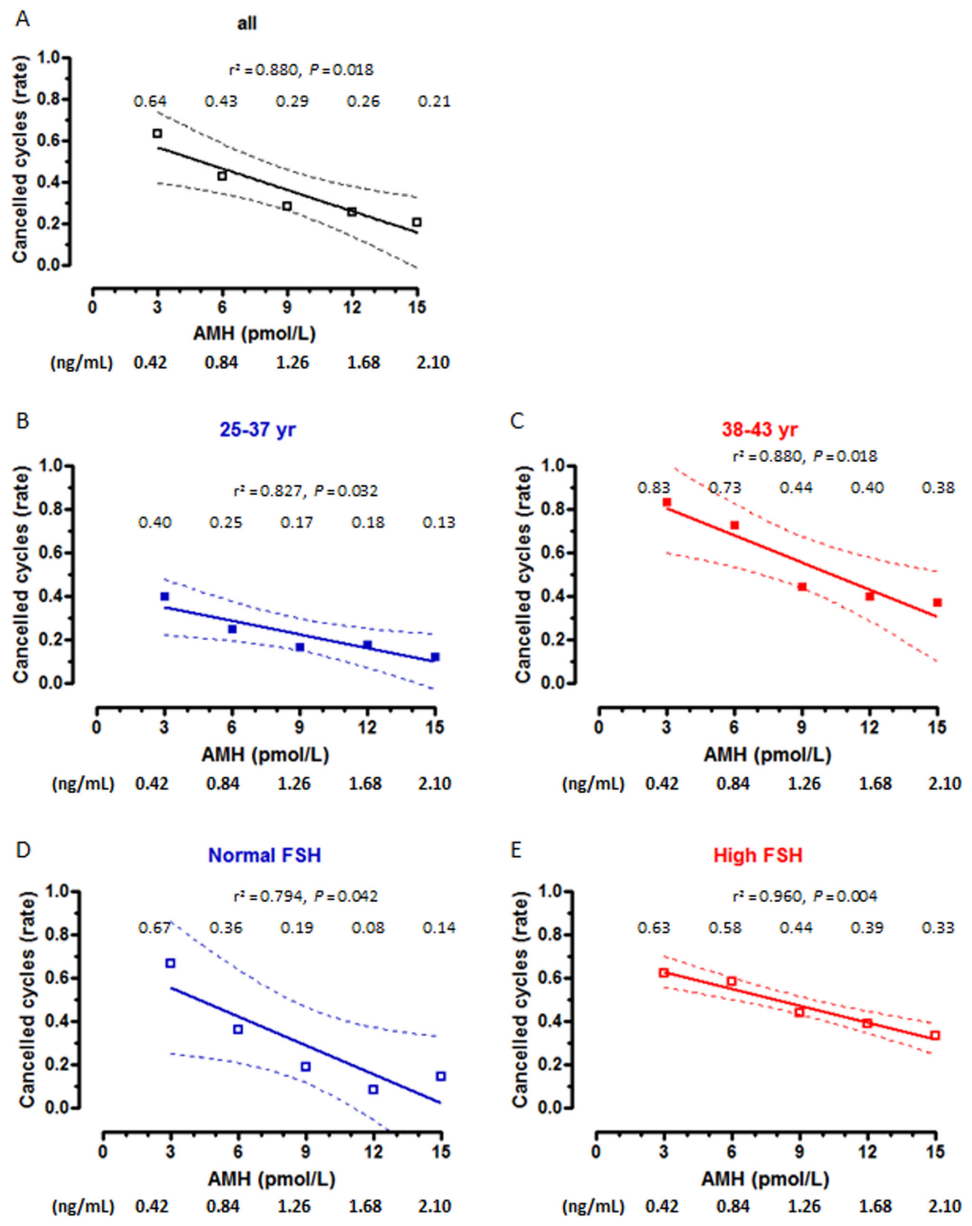
Absolute risk (risk rate) of cancelled cycles as a function of AMH levels. Patients were grouped according to age or FSH levels. Rates are shown for serum AMH at 3, 6, 9, 12 and 15 pmol/L (equivalences for AMH in ng/mL are given below the X axis of each graph). Dotted lines represent the 95% confidence interval.

In the whole group analyzed (Table 1), age and FSH was significantly higher, and AMH was lower in patients with cancelled cycles, as expected. No difference was observed in E2. When patients were grouped by age (Table 2), the significantly lower AMH and higher FSH values were still observed in patients aged 38-43 yr with cancelled cycles. When patients were classified according to their FSH levels (Table 3), lower AMH levels were observed in patients with cancelled cycles irrespective of their FSH levels. Serum AMH showed excellent areas under the ROC curves to predict cycle cancelation in the whole group and in the age and FSH subgroups (Table 4). The absolute risk of cycle cancelation was inversely correlated to serum AMH in the whole group (Figure 1, A) and in the subgroups (Figure 1, B-E). The risk of cycle cancelation in the whole group decreased from 64% in patients with serum AMH ≤3 pmol/L (0.42 ng/mL) to 43% in patients with AMH 6 pmol/L (0.84 ng/mL), 29% with AMH 9 pmol/L (1.26 ng/mL), 26% with AMH 12 pmol/L (1.68 ng/mL) and 21% with AMH ≥15 pmol/L (2.10 ng/mL). When related to patients with AMH <3 pmol/L (0.42 ng/mL), the relative risk of cycle cancelation was 0.42 in patients with AMH between 6-9 pmol/L (0.84-1.26 ng/mL) and 0.37 in patients with AMH >15 pmol/L (>2.1 ng/mL) (Table 5). Interestingly, cycle cancelation occurred in approximately two-thirds of the patients with AMH ≤3 pmol/L irrespective of the FSH level (Figure 1, D and E). However, with higher AMH values the risk of cycle cancelation decreased more significantly in patients with normal FSH.

**Table 1:** Characteristics of the whole study population with cancelled and non-cancelled cycles.

	**Non cancelled**	**Cancelled**	***P***
n	114	31	
Age (yr)	34 (32-38)	38 (36-40)	0.003
AMH (pmol/L)	14.4 (8.6-24.9)	3.7 (<2.5-11.1)	< 0.001
FSH (IU/L)	7.3 (5.9-9.2)	11.4 (7.5-16.8)	0.016
E2 (pg/ml)	47 (29-71)	43 (31-75)	0.871

Results are reported as median and interquartile range.

Comparisons between groups were made using a Mann-Whitney test.

**Table 2:** Characteristics of the whole study population with cancelled and non-cancelled cycles, grouped by age.

	**25-37 yr**			**38-43 yr**		
	**Non cancelled**	**Cancelled**	***P***	**Non cancelled**	**Cancelled**	***P***
n	83	16		31	15	
Age (yr)	33 (31-35)	35 (31-37)	0.250	39 (38-41)	39 (38-40)	0.985
AMH (pmol/L)	14.7 (8.9-27.2)	8.7 (3.5-19.9)	0.071	12.6 (7.5-22.5)	2.5 (<2.5-4.2)	< 0.001
FSH (IU/L)	7.0 (6.1-9.2)	8.7 (7.4-13.8)	0.060	7.8 (5.3-9.4)	13.4 (7.1-22.7)	0.020
E2 (pg/ml)	45 (28-72)	44 (27-76)	0.993	49 (35-72)	43 (33-79)	1.000

Results are reported as median and interquartile range.

Comparisons between groups were made using a Mann-Whitney test.

**Table 3:** Characteristics of the whole study population with cancelled and non-cancelled cycles, grouped by FSH levels.

	**Normal FSH**			**High FSH**		
	**Non cancelled**	**Cancelled**	***P***	**Non cancelled**	**Cancelled**	***P***
N	86	9		28	22	
Age (yr)	34 (31-37)	37 (33-40)	0.246	36 (33-38)	38 (37-39)	0.039
AMH (pmol/L)	16.3 (9.5-26.3)	6.4 (3.3-17.0)	0.017	11.9 (5.5-15.0)	<2.5 (<2.5-5.1)	0.005
FSH (IU/L)	6.7 (5.5-7.9)	7.6 (5.6-9.2)	0.292	10.8 (9.6-13.2)	16.8 (13.7-23.8)	0.004
E2 (pg/ml)	48 (34-75)	71 (33-110)	0.348	44 (23-68)	40 (30-63)	0.922

Results are reported as median and interquartile range.

Comparisons between groups were made using a Mann-Whitney test.

**Table 4:** Areas under the ROC curve (± standard error) for age and hormone levels to assess the value of serum AMH as a predictor of cycle cancelation in the study population.

	**All**	**25-37 yr**	**38-43 yr**	**Normal FSH**	**High FSH**
Age	0.683 ± 0.071	0.623 ± 0.121	0.504 ± 0.100	0.618 ± 0.103	0.753 ± 0.103
AMH	0.813 ± 0.065	0.695 ± 0.108	0.928 ± 0.055	0.744 ± 0.103	0.848 ± 0.080
FSH	0.726 ± 0.075	0.704 ± 0.092	0.765 ± 0.106	0.608 ± 0.102	0.857 ± 0.059
E2	0.514 ± 0.080	0.502 ± 0.108	0.502 ± 0.127	0.626 ± 0.137	0.515 ± 0.109

**Table 5:** Relative risk (RR) of cycle cancelation and good response (RR was considered as 1 in patients with AMH < 3 pmol/L).

	**Cycle cancelation**			**Good response**		
	**RR**	**95%CI**	***P***	**RR**	**95%CI**	***P***
3.0-5.9 pmol/L	0.61	0.24-1.54	0.395	5.00	0.68-36.68	0.155
6.0-8.9 pmol/L	0.42	0.19-0.95	0.012	8.84	1.33-58-89	0.006
9.0-11.9 pmol/L	0.43	0.19-0.99	0.036	8.00	1.17-54.52	0.009
12.0-14.9 pmol/L	0.40	0.18-0.90	0.004	11.33	1.73-74.30	<0.001
≥ 15 pmol/L	0.37	0.17-0.81	<0.001	11.31	1.73-73.99	<0.001

### Oocyte retrieval

**Figure 2:**
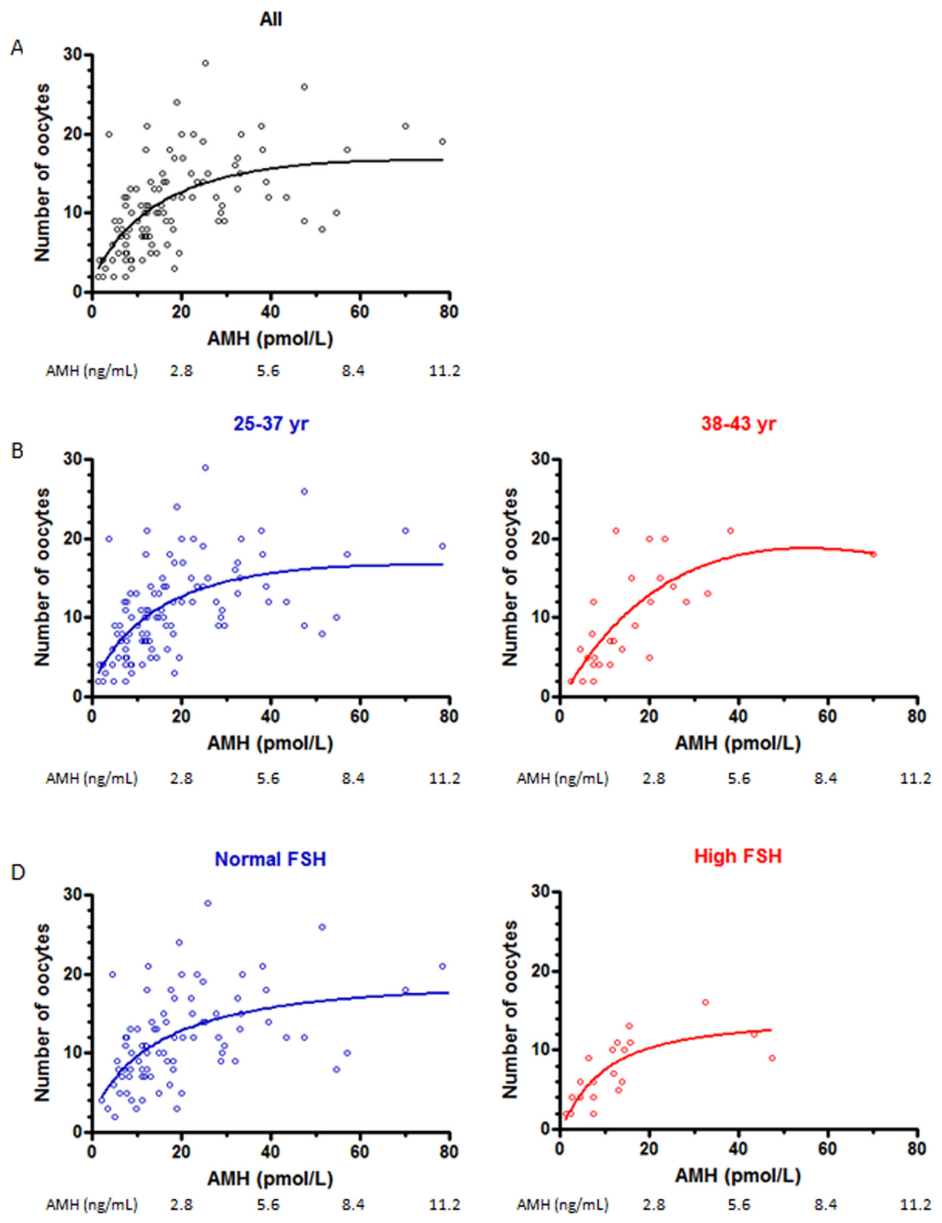
Number of oocytes retrieved at the time of aspiration as a function of AMH levels (non-linear regression). Patients were grouped according to age or FSH levels.

The number of oocytes retrieved in non-cancelled cycles increased progressively in correlation with serum AMH up to an AMH level of 40 pmol/L (5.6 ng/mL) and plateaued thereafter (Figure 2). The increase was observed irrespective of age or serum FSH, yet with a lower absolute number of oocytes in patients aged 38-43 years or with elevated FSH.

Serum AMH showed the most significant differences between patients with good and poor response to stimulation, i.e. retrieval of 5 or more oocytes (Tables 6, 7 and 8). Among basal serum hormone determinations, AMH also showed areas under the ROC curves of high performance to predict a good response (Table 9).

**Table 6:** Age, hormone levels and follicle count in the whole study population with good and poor response to controlled ovarian hyperstimulation.

	**Good response**	**Poor response**	***P***
n	83	31	
Age (yr)	33 (31-37)	37 (34-40)	0.002
AMH (pmol/L)	15.9 (11.3-28.0)	4.6 (2.5-9.8)	< 0.001
FSH (IU/L)	7.3 (5.9-8.7)	10.5 (6.4-16.8)	0.003
E2 (pg/ml)	46 (29-75)	45 (28-70)	0.667
E2-dhCG	1574 (1196-2273)	773 (521-1059)	0.002
Follicle count	7 (5-10)	3 (2-4)	< 0.001

Results are reported as median and interquartile range.

Comparisons between groups were made using a Mann-Whitney test.

**Table 7:** Age, hormone levels and follicle count in patients with good and poor response to controlled ovarian hyperstimulation, grouped by age.

	**25-37 yr**			**38-43 yr**		
	**Good response**	**Poor response**	***P***	**Good response**	**Poor response**	***P***
n	62	16		21	15	
Age (yr)	33 (31-35)	35 (33-37)	0.016	39 (38-40)	40 (38-41)	0.241
AMH (pmol/L)	18.3 (12.1-33.5)	5.8 (2.8-11.1)	< 0.001	16.9 (9.5-24.3)	3.7 (<2.5-7.5)	< 0.001
FSH (IU/L)	7.0 (6.1-8.7)	7.69 (6.0-12.6)	0.102	7.8 (5.3-8.1)	14.5 (6.8-19.0)	0.008
E2 (pg/ml)	45 (29-76)	45 (27-73)	0.825	53 (36-80)	46 (27-66)	0.410
E2-dhCG	1751 (1199-2504)	646 (400-1025)	0.003	1664 (1303-2254)	894 (555-1168)	0.023
Follicle count	8 (5-10)	3 (2-4)	< 0.001	7 (5-9)	3 (2-4)	0.019

Results are reported as median and interquartile range.

Comparisons between groups were made using a Mann-Whitney test.

**Table 8:** Age, hormone levels and follicle count in patients with good and poor response to controlled ovarian hyperstimulation, grouped by FSH levels.

	**Normal FSH**			**High FSH**		
	**Good response**	**Poor response**	***P***	**Good response**	**Poor response**	***P***
n	68	17		15	14	
Age (yr)	31 (33-37)	37 (34-41)	0.029	34 (31-37)	38 (37-39)	0.007
AMH (pmol/L)	16.9 (11.1-28.3)	8.7 (3.6-11.2)	< 0.001	13.2 (11.7-15.7)	2.7 (<2.5-5.8)	< 0.001
FSH (IU/L)	6.7 (5.6-7.9)	6.9 (5.3-7.7)	0.76	10.1 (9.1-10.9)	16.8 (13.2-21.2)	< 0.001
E2 (pg/ml)	46 (32-73)	57 (38-89)	0.337	48 (24-76)	40 (25-49)	0.359
E2-dhCG	1600 (1207-2478)	894 (761-1059)	0.004	1288 (1009-1916)	433 (111-1090)	0.045
Follicle count	8 (6-10)	3 (2-4)	< 0.001	5 (5-10)	3 (2-4)	0.054

Results are reported as median and interquartile range.

Comparisons between groups were made using a Mann-Whitney test.

**Table 9:** Areas under the ROC curve (± standard error) for age and hormone levels to assess the value of serum AMH as a predictor of good response to controlled ovarian hyperstimulation in the study population.

	**All**	**25-37 yr**	**38-43 yr**	**Normal FSH**	**High FSH**
Age	0.683 ± 0.071	0.623 ± 0.121	0.504 ± 0.100	0.669 ± 0.073	0.793 ± 0.085
AMH	0.813 ± 0.065	0.695 ± 0.108	0.928 ± 0.055	0.783 ± 0.065	0.938 ± 0.041
FSH	0.726 ± 0.075	0.704 ± 0.092	0.765 ± 0.106	0.525 ± 0.080	0.976 ± 0.023
E2	0.514 ± 0.080	0.502 ± 0.108	0.502 ± 0.127	0.587 ± 0.088	0.602 ± 0.108

**Figure 3:**
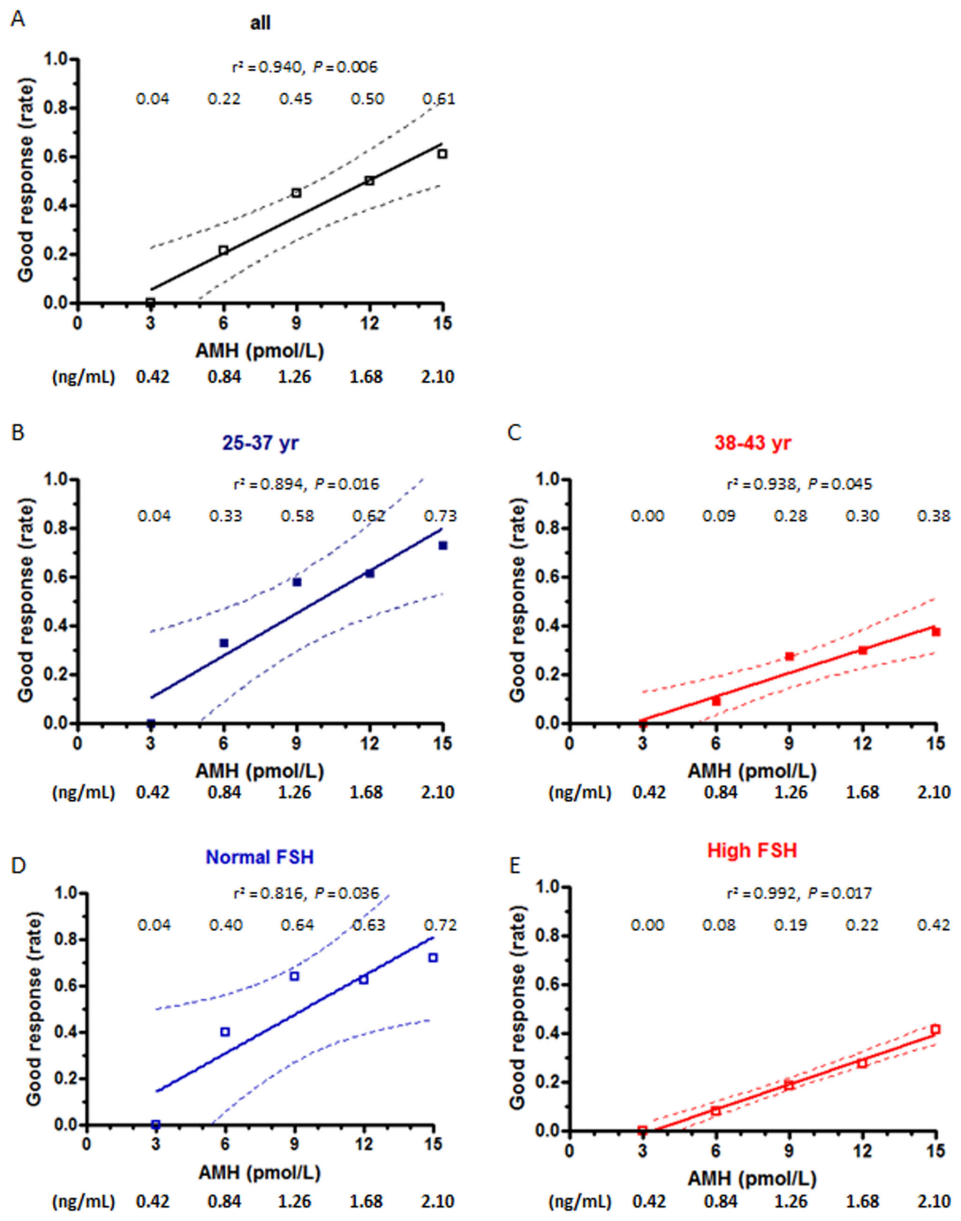
Absolute risk (risk rate) of good response to ovarian stimulation, defined as ≥ 5 oocytes retrieved at the time of aspiration, as a function of AMH levels. Patients were grouped according to age or FSH levels. Rates are shown for serum AMH at 3, 6, 9, 12 and 15 pmol/L (equivalences for AMH in ng/mL are given at the bottom of the figure). Dotted lines represent the 95% confidence interval.

The rate of good response increased with serum AMH: it was almost null in patients with AMH ≤3 pmol/L (0.42 ng/mL) in both age groups (Figure 3) and rose up to 61% in those patients with AMH 15 pmol/L (2.10 ng/mL). When related to patients with AMH <3 pmol/L (0.42 ng/mL), the chances of good response were approximately eight-fold higher in patients with AMH between 6-12 pmol/L (0.84-1.68 ng/mL) and eleven-fold higher in patients with AMH >15 pmol/L (>2.1 ng/mL) (Table 5). The positive correlation between good response and AMH was also significant, but with lower absolute rates, when patients were grouped according to their age (Figure 3, B-C) or FSH levels (Figure 3, D-E).

### Syngamy, cleavage, implantation and pregnancy rates

No significant correlation was found between AMH levels and the rates of syngamy (Spearman r -0.080, *P*=0.313), cleavage at 48 h (Spearman r -0.004, *P*=0.623), multinucleated embryos (Spearman r -0.062, *P*=0.457) or implantation (Spearman r -0.053, *P*=0.557).

**Figure 4:**
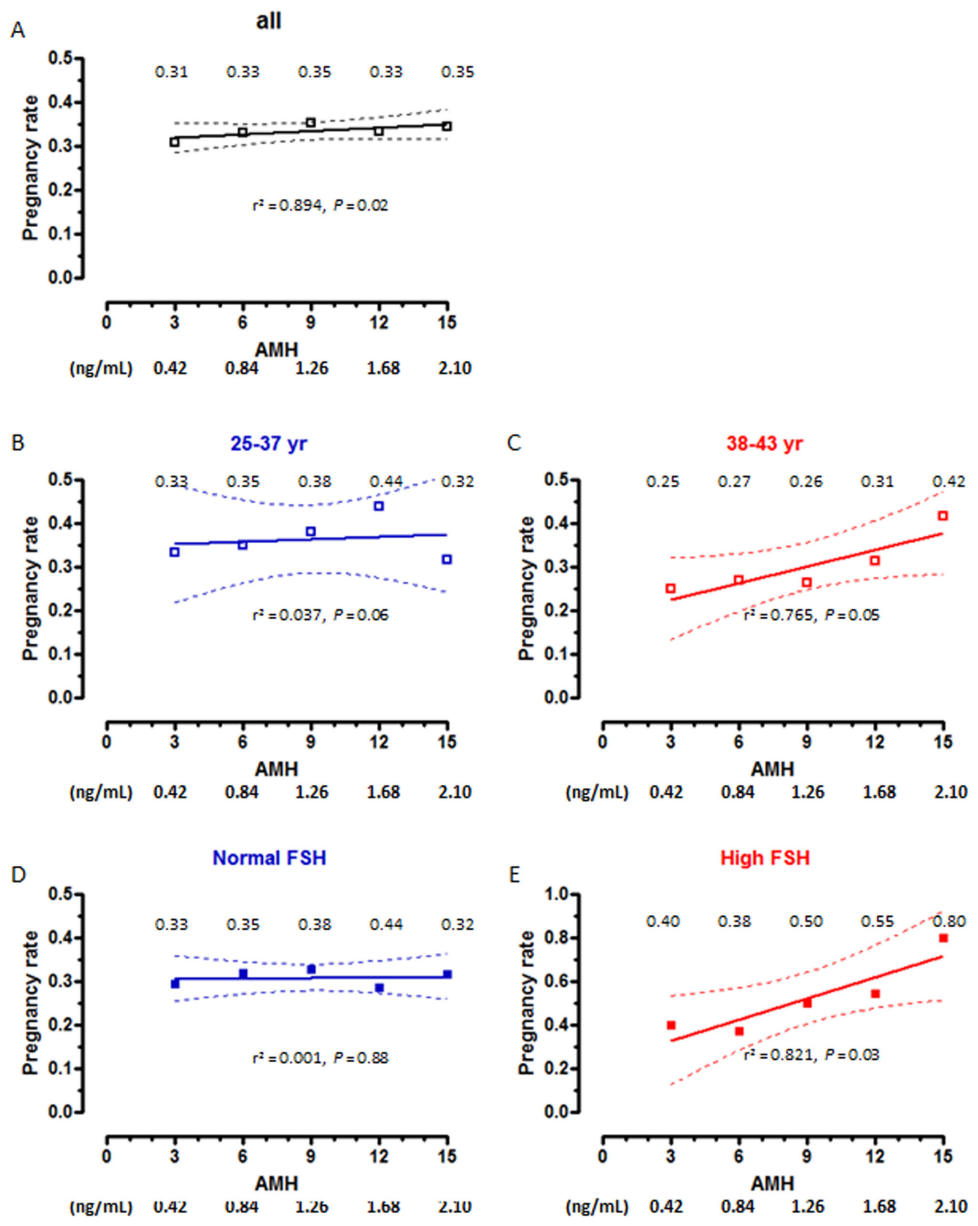
Absolute risk (risk rate) of clinical pregnancy rates as a function of AMH levels. Patients were grouped according to age or FSH levels. Rates are shown for serum AMH at 3, 6, 9, 12 and 15 pmol/L (equivalences for AMH in ng/mL are given at the bottom of the figure). Dotted lines represent the 95% confidence interval.

The rate of pregnancy showed an increase in correlation with serum AMH in the whole group. Serum AMH was lower and age was higher in patients with a higher pregnancy rate when the analysis was performed in the whole group (Table 10). Interestingly, this was also observed in patients with high FSH (Tables 11 and 12). A significantly positive correlation between pregnancy rate and AMH was observed in the whole group (Spearman r0.894, *P*=0.020, Figure 4 A), and in patients >38 yr (Figure 4 C) or with high FSH (Figure 4 E).

**Table 10:** Age, hormone levels and follicle count in the whole study population according to the achievement of clinical pregnancy.

	**Pregnancy**	**Nonpregnancy**	***P***
n	35	89	
Age (yr)	33 (31-37)	36 (33-39)	0.028
AMH (pmol/L)	15.4 (12.0-32.6)	11.6 (5.9-21.5)	0.015
FSH (IU/L)	7.1 (5.2-9.1)	7.4 (6.2-9.7)	0.289
E2 (pg/ml)	47 (29-70)	49 (32-71)	0.964
E2-dhCG	1463 (1021-1944)	1391 (803-2264)	0.936
Follicle count	8 (5-10)	7 (4-9)	0.094

Results are reported as median and interquartile range.

Comparisons between groups were made using a Mann-Whitney test.

**Table 11:** Age, hormone levels and follicle count in patients, grouped by age, according to the achievement of clinical pregnancy.

	**25-37 yr**			**38-43 yr**		
	**Pregnancy**	**Nonpregnancy**	***P***	**Pregnancy**	**Nonpregnancy**	***P***
n	27	59		8	30	
Age (yr)	32 (31-34)	34 (32-36)	0.067	39 (38-41)	40 (39-41)	0.263
AMH (pmol/L)	14.9 (12.1-33.3)	13.9 (7.5-22.8)	0.092	16.4 (7.7-21.9)	7.6 (3.7-15.4)	0.113
FSH (IU/L)	7.6 (5.7-9.3)	6.9 (5.6-8.7)	0.515	5.3 (4.5-7.9)	8.1 (7.1-14.5)	0.015
E2 (pg/ml)	37 (29-67)	50 (33-69)	0.495	64 (49-77)	43 (31-79)	0.135
E2-dhCG	1443 (876-1927)	1411 (923-2467)	0.456	1669 (1285-2229)	1243 (712-1862)	0.171
Follicle count	8 (6-10)	7 (4-10)	0.250	8 (4-10)	5 (3-9)	0.238

Results are reported as median and interquartile range.

Comparisons between groups were made using a Mann-Whitney test.

**Table 12:** Age, hormone levels and follicle count in patients, grouped by FSH levels, according to the achievement of clinical pregnancy.

	**Normal FSH**			**High FSH**		
	**Pregnancy**	**Nonpregnancy**	***P***	**Pregnancy**	**Nonpregnancy**	***P***
n	27	68		8	21	
Age (yr)	34 (31-38)	35 (32-38)	0.513	33 (31-34)	38 (37-39)	< 0.001
AMH (pmol/L)	15.9 (12.0-32.6)	14.7 (7.8-25.1)	0.180	15.0 (7.1-36.6)	5.7 (<2.5-11.9)	0.008
FSH (IU/L)	6.3 (4.8-7.9)	6.7 (5.6-7.8)	0.356	9.9 (9.1-10.9)	13.1 (10.4-18.3)	0.014
E2 (pg/ml)	53 (30-80)	50 (32-75)	0.811	31 (29-64)	45 (27-65)	0.647
E2-dhCG	1463 (944-1950)	1467 (923-2467)	0.523	1586 (1150-2029)	1242 (506-1765)	0.097
Follicle count	8 (6-10)	7 (4-9)	0.112	7 (4-10)	4 (3-10)	0.535

Results are reported as median and interquartile range.

Comparisons between groups were made using a Mann-Whitney test.

## Discussion

One of the most critical aspects before starting an ART procedure is the initial evaluation of the female’s capacity to produce healthy and developmental competent oocytes. Serum AMH has become a standard determination to evaluate the ovarian reserve. In the present study, we provide practical data for the clinician and the couple to easily predict the odds for cycle cancelation, good response to stimulation and pregnancy, according to the level of circulating AMH in a random sample obtained prior to initiating the ART procedure. On the basis of the data provided in Figure 1, the clinician can give practical counseling: risk of cycle cancelation attains approximately two-thirds of the couples if serum AMH is 3 pmol/L, 1/3 if AMH is 9 pmol/L and 1/5 if AMH is 15 pmol/L. Further discrimination can be made according to patient’s age or serum FSH. Similarly, as shown in Figure 3, less than one-twentieth of the attempts will yield a good response to stimulation when serum AMH is 3 pmol/L, but the rate increases to approximately 1/5 when AMH is 6 pmol/L, approximately ½ when AMH is 9-12 pmol/L and almost 2/3 when AMH is 15 pmol/L. Many authors have previously analyzed the association between serum AMH and reproductive outcomes in women undergoing ART treatments, as summarized in a recent systematic review [5]. Most of these studies provide correlation coefficients or sensitivity and specificity levels for a given AMH cutoff, using adequate statistical procedures to avoid confounders. Although the strength of the association has been unequivocally proven, correlation coefficients or more complicate calculations do not represent practical tools for straightforward application in clinical practice. In the present work we have used a practical approach and provide useful and easily applicable results of serum AMH levels to predict the risk of cycle cancelation and the chances of good response to controlled ovarian hyperstimulation and pregnancy in females undergoing an ART procedure, according to their age or FSH level. Some studies have reported predictive values for poor response and pregnancy rates, but only using 1 or 2 cutoff levels of serum AMH [20,27,31–36]. Nelson and colleagues [37] reported more discriminated prediction rates of oocyte retrieval and live births, for five different serum AMH ranges. Here, we provide detailed results for several reproductive outcomes helping to predict the success rate of many steps of the ART procedure. One particularly relevant, from a practical standpoint, is the prediction of the risk of cycle cancelation, which has not been analyzed by previous publications.

Our results are in line with those previously reported by other authors showing that AMH is a useful marker for predicting cycle cancelation [38] and poor response to ovarian stimulation [5], representing a better predictor than the other classically associated parameters such as FSH, estradiol and age [39]. Interestingly, our data show the special importance of serum AMH in women aged >38 yr or with high serum FSH, and have immediate application for the clinician and the patient in their decision prior to start hormonal stimulation. For example, in women 38-43 yr seeking for assisted reproduction treatment, an AMH value ≥ 15 pmol/L (2.10 ng/mL) indicates a risk of 38% to result in a cycle cancelation, and the risk increases to 83% if AMH is ≤ 3 pmol/L (0.42 ng/mL). Also, different AMH values are helpful for decision making in both patients with normal or elevated FSH levels.

It is well established that AMH is correlated with the number of oocytes retrieved at the time of aspiration [40]. Our results are consistent with this observation, independently of the patient’s age. Furthermore, from a practical standpoint, we show that AMH is particularly useful to predict ovarian response to stimulation, as shown by the best areas under the ROC curves. Only E2-dhCG showed a better area under the ROC curve; yet, it cannot be used as a predictor before starting the stimulation protocol.

In our hands, AMH was not as powerful as a predictor of oocyte quality in terms of fertilization rate, embryo development and implantation. Although we found a correlation between serum AMH and pregnancy rate in the whole group, the effect magnitude was modest, in line with the controversial results reported by other groups showing no [41–43] or moderate usefulness of AMH in predicting embryo development [8,44]. However, it was interesting to note that AMH was more discriminant in the high-risk groups, i.e. in the patients >38 yr or with high FSH.

In conclusion, in an individual patient seeking for assisted reproductive technology treatment, the serum AMH level measured in a previous cycle can be used to predict the risk of cycle cancelation and the chances of good ovarian response, when analyzed together with patient’s age or serum FSH. Serum AMH has a significantly better predictive value than FSH and follicle count particularly in women > 38 yr. Furthermore, serum AMH is useful to predict reproductive outcomes in patients with a mild to moderate increase in FSH levels. Although our study design does not allow us to conclude that an infertility treatment should be interrupted on the basis of low AMH, our results add to the existing evidence, and provide practical information, on the usefulness of serum AMH level to help clinicians and patients estimate the chances of good treatment outcomes before initiating the stimulation protocol. AMH evaluation is not routinely performed prior to stimulation and should be incorporated into the initial screening of the patient.
